# Absent MicroRNAs in Different Tissues of Patients with Acquired Cardiomyopathy

**DOI:** 10.1016/j.gpb.2016.04.005

**Published:** 2016-07-28

**Authors:** Christine S. Siegismund, Maria Rohde, Uwe Kühl, Felicitas Escher, Heinz Peter Schultheiss, Dirk Lassner

**Affiliations:** 1 Institute for Cardiac Diagnostics and Therapy (IKDT), 12203 Berlin, Germany; 2 Department of Cardiology, Campus Virchow, Charité – University Hospital Berlin, 13353 Berlin, Germany

**Keywords:** Cardiomyopathy, Heart muscle biopsy, Absent miRNAs, Peripheral blood mononuclear cell, Serum

## Abstract

MicroRNAs (miRNAs) can be found in a wide range of tissues and body fluids, and their specific signatures can be used to determine diseases or predict clinical courses. The miRNA profiles in biological samples (tissue, **serum**, **peripheral blood mononuclear cells** or other body fluids) differ significantly even in the same patient and therefore have their own specificity for the presented condition. Complex profiles of deregulated miRNAs are of high interest, whereas the importance of non-expressed miRNAs was ignored. Since miRNAs regulate gene expression rather negatively, **absent miRNAs** could indicate genes with unaltered expression that therefore are normally expressed in specific compartments or under specific disease situations. For the first time, non-detectable miRNAs in different tissues and body fluids from patients with different diseases (cardiomyopathies, Alzheimer’s disease, bladder cancer, and ocular cancer) were analyzed and compared in this study. miRNA expression data were generated by microarray or TaqMan PCR-based platforms. Lists of **absent miRNAs** of primarily cardiac patients (myocardium, blood cells, and **serum**) were clustered and analyzed for potentially involved pathways using two prediction platforms, *i.e.*, miRNA enrichment analysis and annotation tool (miEAA) and DIANA miRPath. Extensive search in biomedical publication databases for the relevance of non-expressed miRNAs in predicted pathways revealed no evidence for their involvement in heart-related pathways as indicated by software tools, confirming proposed approach.

## Introduction

Cardiovascular diseases as life-threatening diseases are the most common cause of death in Western European countries [1]. Myocarditis and non-ischemic dilated cardiomyopathy (DCM) are acute or chronic disorders of heart muscle which arises mainly from myocardial inflammation or infections by cardiotropic viruses [1], [2], [3], [4], [5], [6]. More than 12 million patients in Europe and 15 million patients in the United States (US) are suffering from heart failure including four million with DCM, according to an estimation of the European Society of Cardiology (ESC) [3]. The traditional clinical diagnosis based on individual patient’s clinical symptoms, medical and family history, laboratory and imaging evaluations should be expanded by endomyocardial biopsy (EMB) diagnostics (virology, histology, and immunohistochemistry) to confirm myocardial disease for following treatment decisions [3], [7], [8].

Improvements in human genetic studies and the continuously-expanding field of biomarker discovery revealed the potential of physiological biomarkers such as microRNAs (miRNAs) or gene expression profiles for diagnosis of complex diseases such as cardiomyopathies and for applications in personalized medicine [9], [10], [11], [12], [13], [14]. miRNA profiling can serve as a new exciting tool in modern diagnostics, which is comparable to gene expression analysis but with less amount of analytes. In addition, approximately 2500 human mature miRNAs have been discovered so far, which seems to be relatively small in number compared to the enormous number of genes discovered [15], [16], [17], [18], [19], [20], [21].

miRNAs are 20–22 nucleotides in length and highly-conserved non-coding RNAs. They have been demonstrated to play multiple roles in negative or positive regulation of gene expression including transcript degradation, translational suppression, or transcriptional and translational activation. miRNAs are present in a wide range of tissues [10], [15], [18], [19], [20], [22], [23], [24], [25], [26]. In body fluids such as serum, plasma or spinal fluid, miRNAs are protected from endogenous RNase activity by inclusion in exosomes or protein complexes [19], [22], [24], [25]. Due to their high biostability, circulating miRNAs can be used as reliable blood-based markers to identify cardiovascular or other human disorders [11], [13], [14], [16], [17], [18], [19].

Up to now, about 800 expressed miRNAs have been experimentally detected in EMBs [21]. As shown for DCM, hypertrophic and inflammatory cardiomyopathy, the expression of miRNAs is characteristically altered in heart tissue [17]. Differential miRNA patterns allow the identification of different heart disorders or disease situations [17], [21]. The role of these human miRNAs in pathogenesis [18] highlights their value as potential molecular biomarkers for complex diseases such as cardiomyopathies [16], [27], [28]. The discriminating power of single miRNAs for diagnosis of complex diseases can be increased by its integration in a larger panel presenting a specific miRNA signature. The application of myocardial miRNA profiling allows the differentiation of distinct phases of viral infections and the prediction of the clinical course of virally-induced disease at the time point of primary diagnostic biopsy [16], [11], [28], [12]. In the same individual, miRNA signatures in tissue, serum, peripheral blood mononuclear cells (PBMCs), or other body fluids show specific features for the current condition. Therefore these disease-specific biomarkers are of increasing interest for personalized medicine [12], [29], [30]. Non-expressed miRNAs in their entirety were ignored and corresponding data were rarely presented [25]. Due to rather negative regulation of miRNAs in general, absent miRNAs would indicate genes which are not altered in terms of expression and therefore normally expressed in specific compartments. Occurrence of previously absent miRNAs could be an easy predictor for changes in functional activity in analyzed biological sample or in the disease situation under examination.

Analyses of expression data by bioinformatic software (miEAA and DIANA [31]) are currently based on two strategies: (1) presentation of published data of deregulated miRNAs and their association with affected pathways or diseases and (2) prediction of involved miRNAs extrapolated from data of differentially expressed genes in corresponding disease situation as presented in the Kyoto Encyclopedia of Genes and Genomes (KEGG) schemata. Comprehensive expression data of indicated pathways or associated disorders are limited by availability of larger patient cohorts and comparability of analytical methods.

In this article, we focused on the non-detectable miRNAs measured on different platforms in myocardial tissue, blood cells, and serum in a large cohort of cardiac patients suffering from different forms of inflammatory or virally-induced heart muscle diseases [1], [2], [3], [4], [5]. The underlying disease was diagnosed by routine EMB [3], [6], [32], [33]. The bioinformatic analyses of generated data using two current freely-available prediction tools revealed no evidence for their involvement in heart-related pathways. Experimental findings for cardiac patients were confirmed by comparisons of absent miRNAs in large cohorts of patients with different diseases [22], [24], [25] measured on the same analytical platforms.

## Results

We performed miRNA expression studies with three analytical platforms, the Geniom Biochips (Febit, Heidelberg, Germany) and two TaqMan PCR-based high-throughput systems including low density array (LDA) and OpenArray (Thermo Fisher Scientific, Waltham, MA, USA). Based on the analysis of deregulated miRNAs, we presented lists and pathways of non-detectable miRNAs in different tissues of primarily cardiac patients. All data were generated in the same laboratory to facilitate comparative data analysis.

### Comparison of absent miRNAs in EMBs, serum, and PBMCs of cardiac patients

**Figure 1:**
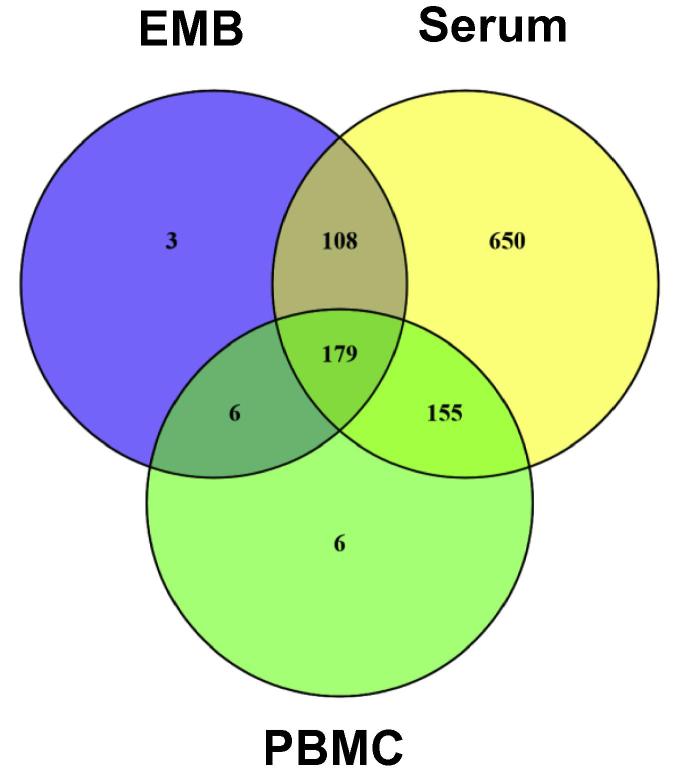
Venn diagram of absent miRNAs in different sample types from cardiac patients Venn diagram analysis was performed for absent miRNAs that are specific to each sample type and overlapping between different sample types such as EMBs (total 296 absent miRNAs), serum (total 1092 absent miRNAs), and PBMCs (total 346 absent miRNAs) of cardiac patients. EMB, endomyocardial biopsy; PBMC, peripheral blood mononuclear cell.

miRNA preparations were obtained for patients with inflammatory or virally-induced cardiomyopathies from EMBs (*n* = 284), PBMCs (*n* = 67), or serum (*n* = 287) including corresponding controls (Table 1). miRNAs in EMBs and serum were measured using two different platforms, which cover different sets of miRNAs (Table 2). Therefore, an additive list for EMBs and serum of absent miRNAs of each system was generated and used for all following calculations. A list of absent miRNAs was generated to indicate common or unique tissues in which miRNAs are not detectable (Table S1). Furthermore, a Venn diagram analysis was performed to reveal overlapping absent miRNAs in EMBs, serum, and PBMCs and miRNAs exclusively absent in particular tissues. As shown in Figure 1, we detected 1107 miRNAs in total absent in 1–3 sample groups. 179 miRNAs were found to be absent in all three sample sources from cardiac patients. The miRNA Enrichment Analysis and Annotation Tool (miEAA) analysis showed that these miRNAs are involved in 685 pathways, implying possibly unaltered genes in these pathways. 7 out of 685 (1.0 %) pathways were indicated to be heart-related. In addition, there are 2 (0.3 %) pathways described for viral myocarditis and DCM. Six miRNAs seem to be associated with these 2 pathways, which include hsa-miR-19b-1-5p, hsa-miR-1295a, hsa-let-7a-5p, hsa-miR-99b-3p, hsa-miR-16-1-3p, and hsa-miR-34b-3p.

On the other hand, some miRNAs are absent only in one sample group. These include 3 miRNAs exclusively absent in EMBs, 6 absent in PBMCs, and 650 absent in serum. For miRNAs absent in EMB or PBMC samples, miEAA revealed 8 pathways but none were heart-related pathways, whereas DIANA miRPath prediction indicated 3 heart-related KEGG pathways for EMBs (57 others) and one for PBMCs (50 others), respectively. For the 650 miRNAs exclusively absent in serum samples, miEAA analysis revealed 14 pathways other than heart-relates ones. Since these patients suffer from cardiac diseases, the missing heart-related pathways are in concordance with the absence of these 650 miRNAs in serum. DIANA miRPath analysis for these miRNAs could not be performed due to limited miRNA input possibility.

### Comparison of absent miRNAs in cardiac patients to those in patients with other diseases

To validate experimental findings for cardiac patients and minimize methodological bias, panels of absent RNAs were evaluated with data from large cohorts of patients with different diseases [22], [24], [25] measured on the same analytical platforms.

**Figure 2:**
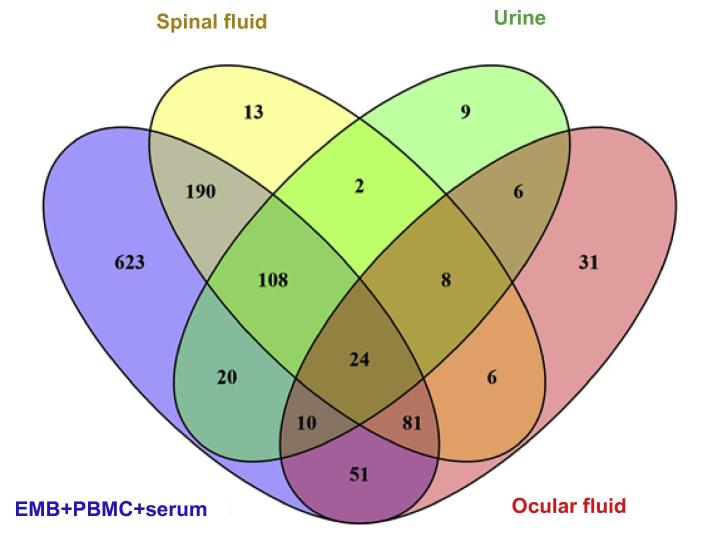
Venn diagram of absent miRNAs in EMBs, PBMCs, and other body fluids Venn diagram analysis was performed for absent miRNAs that are specific to each sample type and overlapping between different sample types. These include EMB, serum, PBMC samples from cardiac patients (total 1107 absent miRNAs), spinal fluid samples from Alzheimer’s disease patients (total 432 absent miRNAs), ocular fluid from ocular cancer patients (total 217 absent miRNAs), and urine from bladder cancer patients (total 187 absent miRNAs). A complete list of the 24 absent miRNAs in all sample types examined and their related pathways are shown in Table 3, Table 4, Table 5, Table 6, Table 7.

We compared the aforementioned 1107 miRNAs absent in any one or more sample groups of EMBs, serum, and PBMCs taken from cardiac patients to those absent in spinal fluid (Alzheimer’s disease patients), urine (bladder cancer patients) or ocular fluid (ocular cancer patients) samples. There are totally 432, 217, and 187 miRNAs absent in spinal fluid, ocular fluid, and urine samples, respectively. Venn diagram showed that 24 absent miRNAs were found to be common among all different tissue types tested. These 24 absent miRNAs were listed in Table 3 [34], [35], [36], [37], [38], [39], [40], [41], [42], [43], [44], [45], [46], [47], [48], [49]. On the other hand, some miRNAs are only absent in one particular group. We found 13, 9, and 31 miRNAs specifically absent in spinal fluid, urine and ocular fluid samples, respectively (Figure 2).

### Pathway comparison using different prediction tools

Next, different pathway prediction tools were employed to analyze the pathways involving the 24 absent miRNAs shared by all samples examined (Table 3). miEAA analysis revealed that these 24 absent miRNAs were involved in only one pathway and in regulation of 10 genes (Table 4) and one disease related to the analyzed miRNAs (Table 5), whereas more than 80 KEGG pathways were predicted with DIANA tool TarBase (Table 6) or microT (Table 7). As shown in Table 4, Table 5, Table 6, Table 7, the number of predicted pathways varied greatly depending on selected prediction algorithm. In addition, the predicted pathways based on the same 24 miRNAs showed associations with completely different diseases or organs using the two software tools. These data raise the question about plausibility and authenticity of the used pathway analysis tools.

## Discussion

The importance of differentially-expressed miRNAs for characterization of various disease situations has been shown impressively [19], [22], [23], [24], [25], [28], [30]. miRNAs are mainly negative regulators of gene expression. Therefore absent miRNAs could indicate genes which are not affected for the disease situation examined or in the corresponding sample material. The different pattern of non-expressed miRNAs in separate tissues or organs could be explained by their biological functions.

The current study described, for the first time, the set of non-expressed miRNAs of the largest published cohort of patients (more than 200, including controls) with inflammatory or virally-induced cardiomyopathies that were diagnosed using EMBs [3], [4], [6], [32]. Absent miRNAs were revealed with different analytical platforms and compared to data from other diseased patients (Alzheimer’s disease, ocular cancer, bladder cancer) measured with identical assays in the same laboratory. The demonstration of differentially regulated miRNAs was not the aim of this study, corresponding data for the differentially-regulated miRNAs were shown previously [16], [22], [24], [25], [28].

Bioinformatic evaluation of identified absent miRNAs was performed by application of two freely-available pathway prediction tools (miEAA and DIANA miRPath) to confirm experimental findings. For cardiac patients, 6 heart-related pathways were recovered using miEAA. For the 6 miRNAs commonly not expressed in EMBs, serum, and PBMCs of cardiac patients, the software predicted association with myocarditis and DCM. Intensive search of biomedical publication databases provided no hint for their involvement in heart muscle diseases. Instead, hsa-miR-16-1-3p is related to chronic lymphocytic leukemia [50] and age-related cataract [51], whereas hsa-miR-34b-3p is related to spermatogenesis [52]. Similarly, hsa-let-7a-5p seems to be related to infectious mononucleosis but not cardiac diseases [45]. Moreover, there lacks proof in literature or through *in silico* prediction tools for the involvement of the remaining 3 miRNAs in any disease or pathway.

DIANA analysis revealed one DCM-related pathway based on the 24 common miRNAs that are never detected in any of EMB, serum, PBMC, spinal fluid, ocular fluid or urine samples. Literature screening in PubMed retrieved no publications related to DCM or other cardiomyopathies for all 24 common absent miRNAs, therefore no experimental proof as well (Table 3, Table 4, Table 5, Table 6, Table 7).

Both examples of detailed search (6 miRNAs and 24 miRNAs) for the relevance of miRNAs in distinct pathways revealed no evidence for their involvement in heart-related pathways as stated in DIANA tool. Pathway prediction tools could generate a broad amount and variety of potential networks which might only exist in theory but not in reality. In addition, these prediction tools have their limits in terms of amount of miRNAs that can be uploaded for analysis (especially DIANA tool), literature evidence of theirs predicted pathways, and comparability between different prediction tools. The best way to overcome this deficiency in pathway prediction is the evaluation of larger sample cohorts or multiple data sources. The involvement of sets of non-expressed miRNAs for more diseases, as presented in this study, will sharpen the predictive power of bioinformatic analyses. These data are easily available but often not requested for publication. In future, predicted pathways should be double checked against list of absent miRNAs. The theoretical output of prediction tools shows high divergence from experimental validation, at least for our study. Therefore, users of prediction tools should take caution and assess the output critically.

The spectrum of non-expressed miRNAs in body fluids for defined diseases such as serum of patients suffering from cardiomyopathies is of keen interest. Today circulating miRNAs have the most important scientific and diagnostic impact [19], [22], [25], [26], [29], [30]. In this article, we described for the first time a panel of absent miRNAs in serum, PBMCs, EMBs, spinal fluid, urine, and ocular fluid of diseased patients including corresponding healthy controls. Implementing this spectrum in comparison to miRNA studies in different disorders, disease-specific miRNAs can be identified expeditiously.

Further studies have to confirm especially which of these absent serum miRNAs in cardiomyopathies are not versatile. Circulating miRNAs will be the novel diagnostic biomarkers, also for heart muscle diseases [14], [15], [21], [24], [26]. Some of these serum miRNAs are present in other disorders not corresponding to cardiomyopathies, which could be of scientific interest for understanding of specific pathomechanisms or finally as therapeutic targets for miRNA modulation to deal with discrete disease situations.

There are some limitations in the current study. Three analytical platforms were used in generating data for overlapping sample sets to infer miRNAs absent alone or in different combinations. EMBs and PBMCs were measured with microarray-based technology for former sets of available miRNAs (miRBase v14), whereas Taqman PCR-based analysis were performed later and used to measure miRNAs in serum (OpenArrays, miRBase v16 and higher), EMBs (LDA and OpenArrays) [15], spinal fluid (OpenArray), urine (LDA), and ocular fluid (LDA). In addition, only two freely-available software tools were used for pathway prediction.

The bioinformatic and translational perspective of presented approach is manifold. This first preliminary study on non-detectable miRNAs should sensitize scientific community to present not only data of deregulated candidates, but also data of completely absent miRNAs [25] as a valuable dataset for improvement of commonly used software tools. Non-detectable miRNAs should be excluded from further prediction of corresponding pathways. Otherwise the collection of these data for all tissues, cells, or body fluids would be an important reservoir for future research or also pharmaceutical studies, and thus should be propagated by bioinformatics. The unexpected finding of previously-described non-expressed miRNAs in an experiment or clinical study will facilitate the identification of newly involved pathways or functional dysregulations in an observed setup.

## Material and methods

### Samples

EMB, PBMC, and serum samples were obtained from healthy controls and patients suffering from inflammatory or virally induced myocarditis as shown in Table 1 [9], [10], [15], [16], [11], [53], [54]. The study was performed within the Transregional Collaborative Research Centre (Inflammatory Cardiomyopathy–Molecular Pathogenesis and Therapy) [Sfb/Tr19]. The study protocol was approved by the local ethics committees of the participating clinical centers, as well as by the committees of the respective federal states. An informed written consent was obtained from each participant.

Spinal fluid samples were received from healthy controls and patients suffering from Alzheimer’s disease, with the ethical statement described previously [25]. Urine samples were acquired from healthy controls and patients harboring bladder cancer, with the ethical statement described previously [22], [24]. In addition, we analyzed pooled ocular fluid from random patients.

### miRNA isolation

miRNAs were obtained from patients, using mirVana™ miRNA Isolation Kit (Thermo Fisher Scientific, Waltham, MA, USA) resp. mirVana™ PARIS™ RNA and Native Protein Purification Kit (Thermo Fisher Scientific, Waltham, MA, USA) for low content samples such as serum, urine, ocular fluid, and spinal fluid according to manufacturer’s instructions. All presented expression studies were performed in the same laboratory.

### miRNA reverse transcription, pre-amplification and expression analysis using TaqMan real-time PCR

Total RNA including miRNA fraction was reversely transcribed to cDNA using Megaplex stem-loop RT primer (Thermo Fisher Scientific, Waltham, MA, USA) for Human Pool A and B in combination with the TaqMan MicroRNA Reverse Transcription Kit (Thermo Fisher Scientific, Waltham, MA, USA). This allowed simultaneous cDNA synthesis of 377 unique miRNAs for Pool A and B each. Except for biopsy materials, a pre-amplification protocol was performed for all low content samples to increase the detection rate. The entire procedure for quantification using TaqMan® OpenArray® [25] and TaqMan® LDA [28] is described elsewhere. miRNAs which were not detectable or above cycle threshold 28 (OpenArrays) resp. 32 (LDA) were considered to be absent in the sample.

### miRNA labeling and expression analysis using Febit Geniom**®** Biochip

The expression analysis of all 906 miRNA and miRNA^∗^ sequences as annotated in Sanger miRBase version 14.0 was performed with the Geniom Real Time Analyzer (Febit, Heidelberg) and the Geniom biochip MPEA hsapiens V14. Sample labeling with biotin was carried out by using the ULS labeling Kit from Kreatech (Amsterdam, The Netherlands). All essential steps such as hybridization, washing, as well as signal amplification and measurement, were done automatically by Geniom Real Time Analyzer. The resulting detection images were evaluated using the Geniom Wizard Software for background correction and normalization of generated data. miRNA expression analyses were carried out using the normalized and background-subtracted intensity values.

### Bioinformatic algorithms and miRNA target identification

miRNAs not detectable in all samples of corresponding biological material were regarded as absent for this material and disease. All following bioinformatics analyses by pathway prediction tools were based on the list of these candidates. Venn diagrams of intersecting sets of miRNAs between different tissues and platforms are generated using Venny v2.0 (http://bioinfogp.cnnb.csic.es/tools/venny/index.html). miEAA (http://www.ccb.uni saarland.de/mieaa_tool) and DIANA miRPath v.2.0 [31] were used for miRNA target prediction and pathway analysis. All given lists of miRNAs are translated and annotated according to miRBase v14 nomenclature.

## Authors’ contributions

CS conducted the bioinformatic algorithms and miRNA target identification, and drafted the manuscript. CS and MR carried out miRNA expression studies. DL conceived the study, and participated in study design and coordination. UK, FE, and HPS had primary responsibility for patient characterization and management. All authors discussed the results, read, and approved the final manuscript.

## Competing interests

The authors declare no competing financial interests or relationships relevant to the content of this paper to disclose.

## Figures and Tables

**Table 1:** Number of analyzed samples sorted by diagnosis and sample type of cardiac patients

**Diagnosis**	**EMB**	**PBMC**	**Serum**
Virally-induced myocarditis total	192	17	166
Adenovirus (ADV)	8		16
Enterovirus (coxsackievirus)	66		72
Human herpes virus 6 (HHV6)		5	12
Chromosomal integrated HHV6 (ciHHV6)		12	13
Parvovirus B19	118		53
Active myocarditis (MCA)	3	8	18
Dilated cardiomyopathy (DCM)	8	6	19
DCM with inflammation (DCMi)	5	7	11
Idiopathic giant cell myocarditis (IGCM)	12	2	8
Amyloidosis	13	4	12
Cardiac sarcoidosis (CS)	6		8
Clinical myocarditis without inflammation (MCno)	4	11	6
Borderline myocarditis (MC-BL)		12	8
Virus-free without inflammation (Vneg)	41		6
Healthy blood donor			25
In total	284	67	287

*Note:* EMB, endomyocardial biopsy; PBMC, peripheral blood mononuclear cell.

**Table 2:** Number of analyzed samples sorted by platform and sample type

**Sample tissue**	**Analyzed samples per miRNA analysis platform**		
**Febit Geniom® Biochip (906 miRNAs)**	**TaqMan® OpenArray® (756–1204 miRNAs)**	**TaqMan® low density array (756 miRNAs)**
EMB	79	137	68
PBMC	67	–	–
Serum	50	237	–
Spinal fluid	–	50	–
Urine	–	–	12
Ocular fluid	–	–	5

*Note:* EMB, endomyocardial biopsy; PBMC, peripheral blood mononuclear cell.

**Table 3:** miRNAs not expressed in any sample type examined in the current study

**miRNA**	**Functional roles**	**Refs.**
hsa-miR-105-5p	NA	
hsa-miR-129-5p	Hepatitis C and hepatocellular carcinoma	[34], [35]
hsa-miR-33b-5p	NA	
hsa-miR-127-5p	NA	
hsa-miR-154-5p	NA	
hsa-miR-199b-5p	Prostate cancer	[36]
hsa-miR-216a-5p	NA	
hsa-miR-216b-5p	NA	
hsa-miR-217	Tumor suppressor for various tumors	[37], [38], [39]
hsa-miR-299-3p	Senescence of endothelial cells	[40]
hsa-miR-330-5p	NA	
hsa-miR-369-3p	Crohn’s disease	[41]
hsa-miR-380-3p	NA	
hsa-miR-98-5p	Hepatitis B	[42]
hsa-miR-122-5p	Hepatitis B	[42], [43]
hsa-miR-147b	NA	
hsa-miR-188-3p	Dendritic plasticity and synaptic transmission	[44]
hsa-miR-18b-5p	Epstein-Barr virus infection	[45]
hsa-miR-198	Retinoblastoma	[46]
hsa-miR-208b-3p	NA	
hsa-miR-339-5p	Lung cancer and oocytogenesis	[47], [48]
hsa-miR-370-3p	NA	
hsa-miR-371a-3p	NA	
hsa-miR-377-3p	Anxiety-related traits	[49]

*Note:* NA means no literature proof found for any disease association with the specified miRNA.

**Table 4:** Overrepresented pathways and genes generated for the 24 commonly-absent miRNAs using miEAA ORA with FDR adjustment

**Category**	**Name of pathway/gene**	***P* value**	**Expected**	**No. of miRNAs involved**
Target pathway	Chromosomal location (Chromosome 14)	0.0002342	0.144668	8
Target gene	*A2M*	0.0351652	0.10582	2
Target gene	*CHST3*	0.0351652	0.0705467	2
Target gene	*FUNDC2*	0.0351652	0.141093	2
Target gene	*MOB3B*	0.0351652	0.141093	2
Target gene	*SLC19A2*	0.0382447	0.176367	2
Target gene	*SMAD7*	0.0351652	0.388007	3
Target gene	*TMEM8A*	0.0382447	0.176367	2
Target gene	*TRAM2*	0.0351652	0.141093	2
Target gene	*TRIB1*	0.0382447	0.176367	2

*Note:* Overrepresented pathways were predicted using miRBase, while target genes regulated by miRNAs were predicted using miRTarBase. miEAA, microRNA enrichment analysis and annotation; ORA, over-representation analysis; FDR, false discovery rate; A2M, alpha-2-macroglobulin; CHST3, carbohydrate sulfotransferase 3; FUNDC2, FUN14 domain containing 2; MOB3B, MOB kinase activator 3B; SLC19A2, solute carrier family 19 member 2; SMAD7, SMAD family member 7; TMEM8A, transmembrane protein 8A; TRAM2, translocation associated membrane protein 2; TRIB1, tribbles pseudokinase 1.

**Table 5:** Predicted diseases by enriched pathways generated for the 24 commonly-absent miRNAs using miEAA G(SEA) with FDR adjustment

**Category**	**Predicted disease**	**Enrichment**	***P* value**	**No. of miRNAs involved**
Diseases	Acute myocardial infarction deregulated	Enriched	0.0411899	10

*Note:* Disease data were based on published studies about miRNA profiles in peripheral blood.

**Table 6:** Pathways generated for the 24 commonly-absent miRNAs using DIANA TarBase

	**KEGG pathway name**	**KEGG pathway ID**	***P* value**	**No. of genes**	**No. of miRNAs**
01	TGF-beta signaling pathway	hsa04350	2.09E-25	44	15
02	ErbB signaling pathway	hsa04012	1.05E-26	46	14
03	Chronic myeloid leukemia	hsa05220	1.11E-25	41	14
04	Ubiquitin mediated proteolysis	hsa04120	6.51E-15	59	16
05	Prostate cancer	hsa05215	9.71E-15	39	15
06	Focal adhesion	hsa04510	3.25E-13	77	16
07	Wnt signaling pathway	hsa04310	7.25E-13	65	15
08	Long-term potentiation	hsa04720	7.77E-13	32	11
09	Glioma	hsa05214	8.35E-13	35	14
10	Dopaminergic synapse	hsa04728	1.65E-12	54	15
11	Non-small cell lung cancer	hsa05223	1.99E-12	27	13
12	Pancreatic cancer	hsa05212	4.78E-12	34	14
13	Melanoma	hsa05218	4.78E-12	32	14
14	Acute myeloid leukemia	hsa05221	3.55E-11	26	15
15	Pathways in cancer	hsa05200	6.73E-12	121	17
16	PI3K-Akt signaling pathway	hsa04151	9.69E-11	114	17
17	Axon guidance	hsa04360	2.76E-10	56	14
18	Renal cell carcinoma	hsa05211	1.03E-09	33	17
19	Prion diseases	hsa05020	8.13E-09	12	12
20	mTOR signaling pathway	hsa04150	1.19E-08	28	12
21	Insulin signaling pathway	hsa04910	1.86E-08	51	16
22	Dorsoventral axis formation	hsa04320	1.89E-08	14	12
23	Bladder cancer	hsa05219	3.31E-08	20	10
24	GnRH signaling pathway	hsa04912	3.61E-08	36	16
25	Hepatitis B	hsa05161	4.18E-09	57	16
26	T cell receptor signaling pathway	hsa04660	5.77E-09	41	15
27	Regulation of actin cytoskeleton	hsa04810	8.72E-08	77	16
28	Fc gamma R-mediated phagocytosis	hsa04666	1.03E-07	37	15
29	GABAergic synapse	hsa04727	2.43E-07	36	14
30	Neurotrophin signaling pathway	hsa04722	2.73E-07	46	17
31	Endometrial cancer	hsa05213	3.52E-07	23	15
32	MAPK signaling pathway	hsa04010	3.65E-07	87	17
33	Nicotine addiction	hsa05033	7.39E-07	21	11
34	Glutamatergic synapse	hsa04724	1.40E-06	46	16
35	HIF-1 signaling pathway	hsa04066	1.97E-06	41	14
36	Small cell lung cancer	hsa05222	2.21E-06	32	13
37	Retrograde endocannabinoid signaling	hsa04723	2.23E-06	44	16
38	Endocytosis	hsa04144	4.98E-07	66	16
39	Colorectal cancer	hsa05210	5.62E-06	26	13
40	Transcriptional misregulation in cancer	hsa05202	8.33E-06	67	17
41	Long-term depression	hsa04730	9.52E-06	30	12
42	HTLV-I infection	hsa05166	1.01E-05	86	18
43	RNA transport	hsa03013	1.03E-05	53	13
44	Gap junction	hsa04540	1.32E-05	36	16
45	Serotonergic synapse	hsa04726	1.86E-05	41	14
46	Shigellosis	hsa05131	1.87E-06	25	13
47	Cholinergic synapse	hsa04725	4.80E-05	44	15
48	Progesterone-mediated oocyte maturation	hsa04914	8.31E-06	31	15
49	B cell receptor signaling pathway	hsa04662	9.67E-05	28	14
50	mRNA surveillance pathway	hsa03015	1.58E-04	32	13
51	Melanogenesis	hsa04916	0.000226175	35	14
52	Adherens junction	hsa04520	0.000287131	34	13
53	p53 signaling pathway	hsa04115	0.000626589	25	14
54	Tight junction	hsa04530	0.000626589	45	17
55	Chemokine signaling pathway	hsa04062	0.000735728	59	17
56	Gastric acid secretion	hsa04971	0.000843576	27	13
57	Thyroid cancer	hsa05216	0.001230756	12	8
58	Aldosterone-regulated sodium reabsorption	hsa04960	0.001230756	15	10
59	Protein processing in endoplasmic reticulum	hsa04141	0.001717442	58	15
60	Fc epsilon RI signaling pathway	hsa04664	0.001997301	25	14
61	Amyotrophic lateral sclerosis	hsa05014	0.002244187	19	12
62	Hedgehog signaling pathway	hsa04340	0.003327063	18	10
63	African trypanosomiasis	hsa05143	0.003397353	13	8
64	RNA degradation	hsa03018	0.003476498	25	12
65	Cell cycle	hsa04110	0.004653399	44	15
66	Hepatitis C	hsa05160	0.005851881	42	15
67	Bacterial invasion of epithelial cells	hsa05100	0.006348145	25	13
68	Vascular smooth muscle contraction	hsa04270	0.007925320	40	17
69	Dilated cardiomyopathy	hsa05414	0.008011601	30	13
70	Circadian rhythm	hsa04710	0.008140682	13	10
71	Tuberculosis	hsa05152	0.011988530	54	16
72	Type II diabetes mellitus	hsa04930	0.012298220	17	10
73	VEGF signaling pathway	hsa04370	0.012587870	22	12
74	Adipocytokine signaling pathway	hsa04920	0.013372720	23	12
75	Steroid biosynthesis	hsa00100	0.017532930	7	6
76	Amoebiasis	hsa05146	0.021327710	35	14
77	Salivary secretion	hsa04970	0.021801720	28	14
78	Hypertrophic cardiomyopathy	hsa05410	0.026359440	27	10
79	Homologous recombination	hsa03440	0.029491120	10	7
80	Fanconi anemia pathway	hsa03460	0.030397850	18	11
81	Chagas disease (American trypanosomiasis)	hsa05142	0.039795790	33	12
82	d-glutamine and d-glutamate metabolism	hsa00471	0.040418440	2	4

**Table 7:** Pathways generated for the 24 commonly-absent miRNAs using DIANA microT

	**KEGG pathway name**	**KEGG pathway ID**	***P* value**	**No. of genes**	**No. of miRNAs**
01	TGF-beta signaling pathway	hsa04350	9.96E-35	43	14
02	Chronic myeloid leukemia	hsa05220	4.60E-28	40	13
03	ErbB signaling pathway	hsa04012	2.74E-18	42	13
04	Prostate cancer	hsa05215	7.11E-13	37	14
05	Pathways in cancer	hsa05200	1.36E-12	116	16
06	Wnt signaling pathway	hsa04310	2.79E-11	60	14
07	Focal adhesion	hsa04510	2.79E-11	71	15
08	Axon guidance	hsa04360	3.33E-11	54	13
09	PI3 K-Akt signaling pathway	hsa04151	3.33E-11	108	17
10	Pancreatic cancer	hsa05212	3.65E-11	32	13
11	Acute myeloid leukemia	hsa05221	4.43E-11	25	14
12	Melanoma	hsa05218	7.58E-12	30	13
13	Non-small cell lung cancer	hsa05223	7.92E-11	25	12
14	Renal cell carcinoma	hsa05211	1.49E-10	32	16
15	Glioma	hsa05214	1.85E-10	32	13
16	Ubiquitin mediated proteolysis	hsa04120	2.01E-10	52	15
17	Fc gamma R-mediated phagocytosis	hsa04666	1.35E-09	37	14
18	Prion diseases	hsa05020	3.26E-09	11	11
19	Long-term potentiation	hsa04720	1.83E-08	28	10
20	Dopaminergic synapse	hsa04728	2.93E-09	47	14
21	Dorso-ventral axis formation	hsa04320	4.36E-08	12	11
22	Small cell lung cancer	hsa05222	5.82E-08	32	12
23	Bladder cancer	hsa05219	1.14E-07	19	9
24	T cell receptor signaling pathway	hsa04660	1.48E-07	39	14
25	Hepatitis B	hsa05161	1.84E-10	54	16
26	Insulin signaling pathway	hsa04910	2.30E-07	47	15
27	Transcriptional misregulation in cancer	hsa05202	2.94E-07	65	16
28	Regulation of actin cytoskeleton	hsa04810	5.00E-07	71	15
29	HIF-1 signaling pathway	hsa04066	1.44E-06	39	13
30	mTOR signaling pathway	hsa04150	2.13E-06	25	11
31	Colorectal cancer	hsa05210	2.70E-06	25	12
32	Neurotrophin signaling pathway	hsa04722	4.60E-06	42	16
33	Endometrial cancer	hsa05213	6.98E-06	21	14
34	Nicotine addiction	hsa05033	9.97E-06	19	10
35	Glutamatergic synapse	hsa04724	1.35E-05	42	15
36	HTLV-I infection	hsa05166	1.57E-05	80	17
37	GABAergic synapse	hsa04727	2.31E-05	32	13
38	B cell receptor signaling pathway	hsa04662	4.03E-05	27	13
39	Adherens junction	hsa04520	4.55E-05	33	12
40	MAPK signaling pathway	hsa04010	5.50E-05	78	16
41	Endocytosis	hsa04144	7.71E-05	60	15
42	Shigellosis	hsa05131	9.06E-05	23	12
43	Serotonergic synapse	hsa04726	0.000134263	38	13
44	GnRH signaling pathway	hsa04912	0.000209828	30	15
45	Thyroid cancer	hsa05216	0.000213265	12	7
46	Aldosterone-regulated sodium reabsorption	hsa04960	0.000213265	15	9
47	p53 signaling pathway	hsa04115	0.000297667	24	13
48	mRNA surveillance pathway	hsa03015	0.000388800	30	12
49	Cholinergic synapse	hsa04725	0.000411066	40	14
50	Progesterone-mediated oocyte maturation	hsa04914	0.000806986	28	14
51	Chemokine signaling pathway	hsa04062	0.000911188	55	16
52	Retrograde endocannabinoid signaling	hsa04723	0.001088369	38	15
53	Cell cycle	hsa04110	0.001246094	42	14
54	Tight junction	hsa04530	0.001246662	42	16
55	Gap junction	hsa04540	0.001678051	31	15
56	VEGF signaling pathway	hsa04370	0.001810046	22	11
57	Amyotrophic lateral sclerosis (ALS)	hsa05014	0.001813055	18	11
58	Bacterial invasion of epithelial cells	hsa05100	0.002374269	24	12
59	Melanogenesis	hsa04916	0.002639431	32	13
60	Hedgehog signaling pathway	hsa04340	0.003261835	17	9
61	Long-term depression	hsa04730	0.004371048	26	11
62	Fc epsilon RI signaling pathway	hsa04664	0.004700800	23	13
63	RNA degradation	hsa03018	0.007308968	23	11
64	African trypanosomiasis	hsa05143	0.007398486	12	7
65	Gastric acid secretion	hsa04971	0.007398486	24	12
66	Dilated cardiomyopathy	hsa05414	0.009484315	28	12
67	Amoebiasis	hsa05146	0.012349140	33	14
68	Tuberculosis	hsa05152	0.013183540	51	16
69	Circadian rhythm	hsa04710	0.014444600	12	9
70	Lysine degradation	hsa00310	0.017716820	16	10
71	Type II diabetes mellitus	hsa04930	0.025168410	16	9
72	D-Glutamine and D-glutamate metabolism	hsa00471	0.026748520	2	3
73	Fanconi anemia pathway	hsa03460	0.026748520	17	10
74	Arrhythmogenic right ventricular cardiomyopathy (ARVC)	hsa05412	0.029365530	26	9
75	RNA transport	hsa03013	0.029365530	44	12
76	Vascular smooth muscle contraction	hsa04270	0.029408600	36	16
77	Protein processing in endoplasmic reticulum	hsa04141	0.030213400	51	14
78	Steroid biosynthesis	hsa00100	0.030239180	6	5
79	Hypertrophic cardiomyopathy (HCM)	hsa05410	0.033928350	25	9
80	Jak-STAT signaling pathway	hsa04630	0.034304140	42	13
81	Chagas disease (American trypanosomiasis)	hsa05142	0.044931410	31	12
82	Homologous recombination	hsa03440	0.046417940	9	6
83	Natural killer cell mediated cytotoxicity	hsa04650	0.048556340	40	13

## Supplementary material

Supplementary Table S1 List of undetectable miRNAs in different tissues and body fluids
