# Supplementary material for: Absent MicroRNAs in Different Tissues of Patients with Acquired Cardiomyopathy
Source: Genomics Proteomics Bioinformatics. 2016 Jul 28;14(4):224–34. doi: 10.1016/j.gpb.2016.04.005 (PMC4996855; doi:10.1016/j.gpb.2016.04.005)
Supplement: Supplementary Table S1 — List of undetectable miRNAs in different tissues and body fluids [file mmc1.docx]

**Table S1 List of undetectable miRNAs in different tissues**

*Note:* "x" means that expression of the specified miRNA is not detetced in the sample examined, while blank input means miRNA expression is detected in the sample examined.

| miRBase ID | miRNAs absent in | | | |
| --- | --- | --- | --- | --- |
|  | **Endomyocrdial biopsy** | **PBMC** | **Serum** | **Spinal fluid** |
| hsa-let-7a-2-3p |  |  | x |  |
| hsa-let-7a-3p |  |  | x | x |
| hsa-let-7a-5p | x | x | x | x |
| hsa-let-7b-3p |  |  | x | x |
| hsa-let-7c-3p |  |  | x | x |
| hsa-let-7c-5p |  |  | x |  |
| hsa-let-7d-3p |  |  |  | x |
| hsa-let-7d-5p |  |  | x |  |
| hsa-let-7e-3p |  |  | x | x |
| hsa-let-7e-5p |  |  | x |  |
| hsa-let-7f-1-3p |  |  | x | x |
| hsa-let-7f-2-3p | x | x | x | x |
| hsa-let-7f-5p |  |  | x |  |
| hsa-let-7g-3p | x |  | x | x |
| hsa-let-7g-5p |  |  | x |  |
| hsa-let-7i-3p | x | x |  | x |
| hsa-let-7i-5p |  |  | x |  |
| hsa-miR-1 |  |  | x |  |
| hsa-miR-100-3p | x | x | x | x |
| hsa-miR-100-5p |  | x | x |  |
| hsa-miR-101-3p |  | x |  |  |
| hsa-miR-101-5p | x | x | x | x |
| hsa-miR-103a-2-5p | x | x | x |  |
| hsa-miR-105-3p |  |  | x | x |
| hsa-miR-105-5p * | x | x | x | x |
| hsa-miR-106a-3p | x | x | x | x |
| hsa-miR-106b-3p | x |  | x | x |
| hsa-miR-107 |  |  |  | x |
| hsa-miR-10a-3p | x | x | x | x |
| hsa-miR-10a-5p |  | x | x |  |
| hsa-miR-10b-3p | x |  | x |  |
| hsa-miR-10b-5p |  | x | x | x |
| hsa-miR-1178 | x |  | x | x |
| hsa-miR-1179 | x | x | x |  |
| hsa-miR-1180 | x | x | x |  |
| hsa-miR-1182 |  |  | x | x |
| hsa-miR-1183 |  |  |  | x |
| hsa-miR-1184 | x | x | x | x |
| hsa-miR-1185-5p | x | x | x |  |
| hsa-miR-1193 |  |  | x |  |
| hsa-miR-1197 | x | x | x | x |
| hsa-miR-1200 | x |  | x | x |
| hsa-miR-1201 |  |  |  | x |
| hsa-miR-1203 |  |  | x | x |
| hsa-miR-1204 | x |  | x | x |
| hsa-miR-1205 | x | x | x | x |
| hsa-miR-1206 | x | x | x | x |
| hsa-miR-1208 |  |  | x |  |
| hsa-miR-122-3p |  |  | x | x |
| hsa-miR-1224-3p | x |  | x | x |
| hsa-miR-1225-3p |  |  | x | x |
| hsa-miR-1225-5p |  |  | x |  |
| hsa-miR-122-5p * |  |  | x | x |
| hsa-miR-1226-3p |  |  | x |  |
| hsa-miR-1226-5p |  |  |  | x |
| hsa-miR-1227-3p |  |  | x |  |
| hsa-miR-1228-3p |  |  | x | x |
| hsa-miR-1228-5p | x |  | x | x |
| hsa-miR-1231 | x |  | x |  |
| hsa-miR-1233 |  |  | x | x |
| hsa-miR-1236 |  |  | x | x |
| hsa-miR-1237-3p |  |  | x |  |
| hsa-miR-1243 | x | x | x |  |
| hsa-miR-1244 | x | x | x |  |
| hsa-miR-1245a | x | x | x | x |
| hsa-miR-124-5p | x | x | x | x |
| hsa-miR-1246 |  |  | x |  |
| hsa-miR-1247-5p | x |  | x | x |
| hsa-miR-1248 |  |  | x | x |
| hsa-miR-1249 |  |  |  | x |
| hsa-miR-1250-5p | x |  | x | x |
| hsa-miR-1251-5p | x | x | x | x |
| hsa-miR-1252 |  |  | x | x |
| hsa-miR-1253 |  |  | x | x |
| hsa-miR-1254 |  |  | x |  |
| hsa-miR-1255a |  |  | x | x |
| hsa-miR-1255b-5p |  |  | x | x |
| hsa-miR-1256 | x |  | x | x |
| hsa-miR-1257 | x |  | x | x |
| hsa-miR-1259 | x | x | x | x |
| hsa-miR-125a-3p |  |  | x |  |
| hsa-miR-125a-5p |  |  | x |  |
| hsa-miR-125b-1-3p | x | x | x | x |
| hsa-miR-125b-2-3p |  |  | x | x |
| hsa-miR-125b-5p |  |  | x |  |
| hsa-miR-1260a | x |  | x |  |
| hsa-miR-1262 | x | x | x |  |
| hsa-miR-1263 | x | x | x | x |
| hsa-miR-1264 | x | x | x | x |
| hsa-miR-1265 | x |  | x | x |
| hsa-miR-126-5p |  | x | x |  |
| hsa-miR-1266-3p | x |  | x |  |
| hsa-miR-1267 |  |  | x |  |
| hsa-miR-1269a | x | x | x |  |
| hsa-miR-1270 |  |  | x | x |
| hsa-miR-1271-5p |  |  | x |  |
| hsa-miR-1272 | x |  | x | x |
| hsa-miR-1273c |  |  | x |  |
| hsa-miR-1273d |  |  | x |  |
| hsa-miR-1273e |  |  | x |  |
| hsa-miR-127-3p |  | x | x |  |
| hsa-miR-1274a |  |  | x |  |
| hsa-miR-1274b |  |  | x |  |
| hsa-miR-1275 |  |  | x | x |
| hsa-miR-127-5p * |  | x | x | x |
| hsa-miR-1276 |  |  | x | x |
| hsa-miR-1278 | x | x | x | x |
| hsa-miR-1279 | x | x | x |  |
| hsa-miR-1282 | x |  | x | x |
| hsa-miR-1283 | x | x | x | x |
| hsa-miR-1284 | x |  | x | x |
| hsa-miR-1285 |  |  |  | x |
| hsa-miR-1286 | x |  | x | x |
| hsa-miR-1287-5p | x | x | x |  |
| hsa-miR-1288 |  |  | x | x |
| hsa-miR-1289 | x |  | x | x |
| hsa-miR-128b | x |  |  |  |
| hsa-miR-1290 |  |  | x |  |
| hsa-miR-1291 |  |  | x |  |
| hsa-miR-129-1-3p |  |  | x |  |
| hsa-miR-1292 |  |  | x | x |
| hsa-miR-1293 |  |  | x | x |
| hsa-miR-129-3p |  |  | x |  |
| hsa-miR-1294 | x |  | x | x |
| hsa-miR-1295a | x | x | x |  |
| hsa-miR-129-5p * | x |  | x | x |
| hsa-miR-1296 |  |  | x | x |
| hsa-miR-1298 | x | x | x |  |
| hsa-miR-1299 |  |  | x |  |
| hsa-miR-1301 | x | x | x | x |
| hsa-miR-1302 |  | x | x | x |
| hsa-miR-1303 |  | x | x |  |
| hsa-miR-1304 | x |  | x | x |
| hsa-miR-1305 | x | x | x | x |
| hsa-miR-1306-3p | x |  | x |  |
| hsa-miR-130a-5p | x | x | x | x |
| hsa-miR-130b-5p |  |  | x | x |
| hsa-miR-1321 |  |  | x |  |
| hsa-miR-1322 | x |  | x |  |
| hsa-miR-1323 |  |  | x |  |
| hsa-miR-132-3p |  |  | x |  |
| hsa-miR-1324 | x |  | x | x |
| hsa-miR-132-5p | x | x | x | x |
| hsa-miR-133a |  |  | x |  |
| hsa-miR-133b |  |  | x |  |
| hsa-miR-134-5p |  |  |  | x |
| hsa-miR-135a-3p |  |  | x |  |
| hsa-miR-135a-5p |  | x | x |  |
| hsa-miR-135b-3p |  | x | x | x |
| hsa-miR-135b-5p |  |  | x |  |
| hsa-miR-136-3p |  |  | x |  |
| hsa-miR-136-5p |  |  | x | x |
| hsa-miR-138-1-3p |  |  | x |  |
| hsa-miR-138-2-3p | x |  | x | x |
| hsa-miR-138-5p |  | x | x |  |
| hsa-miR-139-3p |  |  | x | x |
| hsa-miR-139-5p |  | x | x |  |
| hsa-miR-140-5p |  | x | x |  |
| hsa-miR-141-3p |  | x | x |  |
| hsa-miR-141-5p | x | x | x |  |
| hsa-miR-142-3p |  | x | x |  |
| hsa-miR-142-5p |  |  | x |  |
| hsa-miR-143-5p | x | x | x | x |
| hsa-miR-144-3p | x |  | x |  |
| hsa-miR-144-5p | x |  | x |  |
| hsa-miR-145-3p | x | x | x |  |
| hsa-miR-145-5p |  |  | x |  |
| hsa-miR-1468-5p | x | x | x |  |
| hsa-miR-146a-3p | x | x | x | x |
| hsa-miR-146a-5p |  |  | x |  |
| hsa-miR-146b-3p |  | x | x |  |
| hsa-miR-146b-5p |  |  | x |  |
| hsa-miR-147a | x | x | x | x |
| hsa-miR-147b * |  |  | x | x |
| hsa-miR-148a-3p |  | x |  |  |
| hsa-miR-148a-5p | x |  | x | x |
| hsa-miR-148b-5p | x | x | x |  |
| hsa-miR-149-3p |  |  | x | x |
| hsa-miR-149-5p |  |  | x |  |
| hsa-miR-150-3p |  |  | x |  |
| hsa-miR-150-5p |  |  | x |  |
| hsa-miR-151a-3p |  |  | x |  |
| hsa-miR-152 |  | x | x |  |
| hsa-miR-153-3p |  |  |  | x |
| hsa-miR-1537-3p | x | x | x |  |
| hsa-miR-1538 | x |  | x |  |
| hsa-miR-1539 |  |  | x |  |
| hsa-miR-154-3p | x | x | x | x |
| hsa-miR-154-5p * |  | x | x | x |
| hsa-miR-155-3p |  | x | x | x |
| hsa-miR-155-5p |  | x | x |  |
| hsa-miR-15a-3p | x | x | x | x |
| hsa-miR-15b-3p | x |  | x | x |
| hsa-miR-16-1-3p | x | x | x | x |
| hsa-miR-16-2-3p | x | x | x | x |
| hsa-miR-17-3p |  | x |  | x |
| hsa-miR-181a-2-3p |  | x | x |  |
| hsa-miR-181a-3p |  |  | x |  |
| hsa-miR-181a-5p | x |  | x |  |
| hsa-miR-181c-3p | x |  | x | x |
| hsa-miR-181c-5p |  |  | x |  |
| hsa-miR-182-3p |  | x | x | x |
| hsa-miR-1825 |  |  | x | x |
| hsa-miR-182-5p |  |  | x | x |
| hsa-miR-1826 | x |  | x | x |
| hsa-miR-1827 |  | x | x |  |
| hsa-miR-183-3p |  |  | x | x |
| hsa-miR-183-5p |  | x | x | x |
| hsa-miR-184 |  |  | x |  |
| hsa-miR-185-3p | x |  | x | x |
| hsa-miR-186-3p | x | x | x | x |
| hsa-miR-186-5p |  | x | x |  |
| hsa-miR-187-3p |  | x | x | x |
| hsa-miR-188-3p * |  |  | x | x |
| hsa-miR-188-5p | x | x | x |  |
| hsa-miR-18a-3p | x |  | x | x |
| hsa-miR-18a-5p |  |  | x | x |
| hsa-miR-18b-3p | x |  | x | x |
| hsa-miR-18b-5p * |  |  | x | x |
| hsa-miR-1909-3p |  |  | x |  |
| hsa-miR-1909-5p |  |  | x |  |
| hsa-miR-190a-5p |  | x | x |  |
| hsa-miR-190b | x | x | x |  |
| hsa-miR-1911-3p | x |  | x |  |
| hsa-miR-1911-5p | x | x | x |  |
| hsa-miR-1912 | x |  | x |  |
| hsa-miR-191-3p | x | x | x | x |
| hsa-miR-1914-5p |  |  | x |  |
| hsa-miR-1915-5p | x |  | x |  |
| hsa-miR-192-3p |  |  | x | x |
| hsa-miR-192-5p |  |  | x |  |
| hsa-miR-193a-3p |  |  | x | x |
| hsa-miR-193a-5p |  |  | x |  |
| hsa-miR-193b-3p |  |  | x |  |
| hsa-miR-193b-5p |  |  | x | x |
| hsa-miR-194-3p | x |  | x | x |
| hsa-miR-194-5p |  |  | x |  |
| hsa-miR-195-3p | x |  | x | x |
| hsa-miR-196a | x |  |  |  |
| hsa-miR-196a-3p | x |  | x | x |
| hsa-miR-196b-3p |  |  | x |  |
| hsa-miR-196b-5p |  | x | x |  |
| hsa-miR-1972 |  |  | x |  |
| hsa-miR-1976 |  |  | x |  |
| hsa-miR-198 * |  |  | x | x |
| hsa-miR-199a-3p |  | x | x |  |
| hsa-miR-199a-5p |  |  | x |  |
| hsa-miR-199b-3p |  | x | x |  |
| hsa-miR-199b-5p * |  | x | x | x |
| hsa-miR-19a-5p | x | x | x | x |
| hsa-miR-19b-1-5p | x | x | x | x |
| hsa-miR-19b-2-5p | x | x | x |  |
| hsa-miR-200a-3p |  | x | x | x |
| hsa-miR-200a-5p |  | x | x | x |
| hsa-miR-200b-3p |  | x | x |  |
| hsa-miR-200b-5p | x | x | x | x |
| hsa-miR-200c-3p |  | x | x | x |
| hsa-miR-200c-5p | x |  | x | x |
| hsa-miR-202-3p |  |  | x |  |
| hsa-miR-202-5p |  | x | x | x |
| hsa-miR-203a |  | x | x |  |
| hsa-miR-203b-5p |  |  | x |  |
| hsa-miR-204-5p |  |  | x |  |
| hsa-miR-2052 | x | x | x |  |
| hsa-miR-2053 | x | x | x |  |
| hsa-miR-205-3p |  |  | x |  |
| hsa-miR-205-5p | x |  | x |  |
| hsa-miR-206 |  |  | x |  |
| hsa-miR-208a-3p |  |  | x | x |
| hsa-miR-208b-3p * |  |  | x | x |
| hsa-miR-20a-3p | x | x | x | x |
| hsa-miR-20b-3p | x | x | x | x |
| hsa-miR-20b-5p |  |  | x |  |
| hsa-miR-210-3p |  |  | x | x |
| hsa-miR-2110 |  |  | x |  |
| hsa-miR-2113 | x |  | x |  |
| hsa-miR-2114-5p | x |  | x |  |
| hsa-miR-2115-3p |  | x | x |  |
| hsa-miR-2115-5p | x | x | x |  |
| hsa-miR-211-5p |  |  | x | x |
| hsa-miR-2116-5p | x | x | x |  |
| hsa-miR-2117 | x | x | x |  |
| hsa-miR-212-3p |  |  | x |  |
| hsa-miR-21-3p | x |  | x |  |
| hsa-miR-214-5p | x |  | x |  |
| hsa-miR-215-5p |  |  | x |  |
| hsa-miR-21-5p |  |  | x |  |
| hsa-miR-216a-5p * |  | x | x | x |
| hsa-miR-216b-5p * |  | x |  | x |
| hsa-miR-217 * |  | x | x | x |
| hsa-miR-218-1-3p | x |  | x | x |
| hsa-miR-218-2-3p | x | x | x | x |
| hsa-miR-218-5p |  | x | x |  |
| hsa-miR-219a-1-3p |  | x | x |  |
| hsa-miR-219a-2-3p | x |  | x | x |
| hsa-miR-219a-5p |  |  | x |  |
| hsa-miR-221-3p |  |  | x |  |
| hsa-miR-221-5p ^a^ | x | x | x | x |
| hsa-miR-222-3p |  |  | x |  |
| hsa-miR-222-5p | x |  | x | x |
| hsa-miR-223-5p | x | x | x |  |
| hsa-miR-22-3p |  |  |  | x |
| hsa-miR-224-3p |  | x | x |  |
| hsa-miR-224-5p |  | x | x |  |
| hsa-miR-22-5p |  | x | x | x |
| hsa-miR-2277-3p |  |  | x |  |
| hsa-miR-2277-5p |  |  | x |  |
| hsa-miR-2278 | x |  | x |  |
| hsa-miR-23a-3p |  |  |  | x |
| hsa-miR-23a-5p |  |  | x |  |
| hsa-miR-23b-3p |  |  |  | x |
| hsa-miR-23b-5p | x | x | x | x |
| hsa-miR-23c |  |  | x |  |
| hsa-miR-24-1-5p | x | x | x | x |
| hsa-miR-24-2-5p |  |  | x | x |
| hsa-miR-25-5p |  |  | x | x |
| hsa-miR-26a-1-3p | x | x | x | x |
| hsa-miR-26a-2-3p | x | x | x |  |
| hsa-miR-26b-3p |  |  | x |  |
| hsa-miR-26b-5p |  |  | x |  |
| hsa-miR-27a-3p |  |  | x |  |
| hsa-miR-27a-5p | x | x | x |  |
| hsa-miR-27b-3p |  |  | x |  |
| hsa-miR-27b-5p | x | x | x |  |
| hsa-miR-28-3p |  | x | x |  |
| hsa-miR-28-5p |  |  | x |  |
| hsa-miR-2909 |  |  | x |  |
| hsa-miR-296-3p | x |  | x | x |
| hsa-miR-298 |  |  | x | x |
| hsa-miR-299-3p * |  | x | x | x |
| hsa-miR-299-5p |  |  | x | x |
| hsa-miR-29a-3p |  |  | x |  |
| hsa-miR-29a-5p | x | x | x |  |
| hsa-miR-29b-1-5p | x | x | x | x |
| hsa-miR-29b-2-5p | x | x | x |  |
| hsa-miR-29b-3p |  |  | x |  |
| hsa-miR-29c-3p | x |  | x |  |
| hsa-miR-300 |  |  | x |  |
| hsa-miR-301a-3p |  |  | x |  |
| hsa-miR-302a-3p |  |  | x |  |
| hsa-miR-302a-5p | x | x | x | x |
| hsa-miR-302b-3p |  |  | x | x |
| hsa-miR-302b-5p ^a^ | x | x | x | x |
| hsa-miR-302c-3p |  | x | x |  |
| hsa-miR-302c-5p | x | x | x |  |
| hsa-miR-302d-3p |  |  | x | x |
| hsa-miR-302d-5p | x | x | x | x |
| hsa-miR-302e | x |  | x |  |
| hsa-miR-302f | x |  | x |  |
| hsa-miR-3065-3p |  |  | x |  |
| hsa-miR-3065-5p |  |  | x |  |
| hsa-miR-3074-3p |  |  | x |  |
| hsa-miR-30a-3p |  | x | x |  |
| hsa-miR-30a-5p |  |  | x |  |
| hsa-miR-30b-3p |  |  | x | x |
| hsa-miR-30b-5p |  |  | x |  |
| hsa-miR-30c-1-3p |  |  | x | x |
| hsa-miR-30c-2-3p |  |  | x | x |
| hsa-miR-30c-5p |  |  | x |  |
| hsa-miR-30d-3p | x | x | x |  |
| hsa-miR-30d-5p |  |  | x |  |
| hsa-miR-30e-3p |  | x | x |  |
| hsa-miR-30e-5p |  |  | x |  |
| hsa-miR-3115 |  |  | x |  |
| hsa-miR-3116 |  |  | x |  |
| hsa-miR-3117-3p |  |  | x |  |
| hsa-miR-3117-5p |  |  | x |  |
| hsa-miR-3118 |  |  | x |  |
| hsa-miR-3119 |  |  | x |  |
| hsa-miR-3121-3p |  |  | x |  |
| hsa-miR-3122 |  |  | x |  |
| hsa-miR-3124-3p |  |  | x |  |
| hsa-miR-3126 |  |  | x |  |
| hsa-miR-3126-5p |  |  | x |  |
| hsa-miR-3127-5p |  |  | x |  |
| hsa-miR-3128 |  |  | x |  |
| hsa-miR-3129-5p |  |  | x |  |
| hsa-miR-3131 |  |  | x |  |
| hsa-miR-3132 |  |  | x |  |
| hsa-miR-3133 |  |  | x |  |
| hsa-miR-3134 |  |  | x |  |
| hsa-miR-3135b |  |  | x |  |
| hsa-miR-3136-5p |  |  | x |  |
| hsa-miR-3137 |  |  | x |  |
| hsa-miR-3138 |  |  | x |  |
| hsa-miR-3139 |  |  | x |  |
| hsa-miR-31-3p | x | x |  |  |
| hsa-miR-3140-3p |  |  | x |  |
| hsa-miR-3142 |  |  | x |  |
| hsa-miR-3143 |  |  | x |  |
| hsa-miR-3144-3p |  |  | x |  |
| hsa-miR-3144-5p |  |  | x |  |
| hsa-miR-3145-3p |  |  | x |  |
| hsa-miR-3147 |  |  | x |  |
| hsa-miR-3148 |  |  | x |  |
| hsa-miR-3150a-3p |  |  | x |  |
| hsa-miR-3150b-3p |  |  | x |  |
| hsa-miR-3151-5p |  |  | x |  |
| hsa-miR-3152-3p |  |  | x |  |
| hsa-miR-3153 |  |  | x |  |
| hsa-miR-3154 |  |  | x |  |
| hsa-miR-3155a |  |  | x |  |
| hsa-miR-3156-5p |  |  | x |  |
| hsa-miR-3157-5p |  |  | x |  |
| hsa-miR-3158-3p |  |  | x |  |
| hsa-miR-3159 |  |  | x |  |
| hsa-miR-31-5p |  | x | x |  |
| hsa-miR-3160-3p |  |  | x |  |
| hsa-miR-3161 |  |  | x |  |
| hsa-miR-3162-3p |  |  | x |  |
| hsa-miR-3163 |  |  | x |  |
| hsa-miR-3164 |  |  | x |  |
| hsa-miR-3165 |  |  | x |  |
| hsa-miR-3166 |  |  | x |  |
| hsa-miR-3167 |  |  | x |  |
| hsa-miR-3169 |  |  | x |  |
| hsa-miR-3170 |  |  | x |  |
| hsa-miR-3171 |  |  | x |  |
| hsa-miR-3173-5p |  |  | x |  |
| hsa-miR-3174 |  |  | x |  |
| hsa-miR-3175 |  |  | x |  |
| hsa-miR-3176 |  |  | x |  |
| hsa-miR-3179 |  |  | x |  |
| hsa-miR-3180 |  |  | x |  |
| hsa-miR-3180-3p |  |  | x |  |
| hsa-miR-3181 |  |  | x |  |
| hsa-miR-3182 |  |  | x |  |
| hsa-miR-3183 |  |  | x |  |
| hsa-miR-3184-5p |  |  | x |  |
| hsa-miR-3185 |  |  | x |  |
| hsa-miR-3186-3p |  |  | x |  |
| hsa-miR-3186-5p |  |  | x |  |
| hsa-miR-3187-3p |  |  | x |  |
| hsa-miR-3187-5p |  |  | x |  |
| hsa-miR-3188 |  |  | x |  |
| hsa-miR-3189-3p |  |  | x |  |
| hsa-miR-3190-3p |  |  | x |  |
| hsa-miR-3191-3p |  |  | x |  |
| hsa-miR-3192-5p |  |  | x |  |
| hsa-miR-3193 |  |  | x |  |
| hsa-miR-3195 |  |  | x |  |
| hsa-miR-3197 |  |  | x |  |
| hsa-miR-3198 |  |  | x |  |
| hsa-miR-3199 |  |  | x |  |
| hsa-miR-3200-3p |  |  | x |  |
| hsa-miR-3200-5p |  |  | x |  |
| hsa-miR-3201 |  |  | x |  |
| hsa-miR-3202 |  |  | x |  |
| hsa-miR-320b |  |  | x |  |
| hsa-miR-320c |  |  | x |  |
| hsa-miR-320d |  |  | x |  |
| hsa-miR-320e |  |  | x |  |
| hsa-miR-323-5p |  |  | x |  |
| hsa-miR-323a-3p |  |  | x |  |
| hsa-miR-323b-3p |  |  | x |  |
| hsa-miR-323b-5p | x |  | x | x |
| hsa-miR-32-3p | x | x | x | x |
| hsa-miR-324-3p |  |  | x |  |
| hsa-miR-324-5p |  |  | x |  |
| hsa-miR-325 |  | x | x | x |
| hsa-miR-32-5p |  | x | x |  |
| hsa-miR-326 |  |  | x |  |
| hsa-miR-328 |  |  | x |  |
| hsa-miR-329 |  |  | x | x |
| hsa-miR-330-5p * |  | x | x | x |
| hsa-miR-331-3p |  |  | x |  |
| hsa-miR-331-5p |  | x | x |  |
| hsa-miR-335-3p | x | x | x | x |
| hsa-miR-335-5p |  |  | x |  |
| hsa-miR-337-3p |  | x | x | x |
| hsa-miR-337-5p |  | x | x |  |
| hsa-miR-338-3p |  | x | x |  |
| hsa-miR-338-5p | x | x | x |  |
| hsa-miR-339-5p * |  |  | x | x |
| hsa-miR-33a-3p |  |  | x |  |
| hsa-miR-33a-5p | x | x | x | x |
| hsa-miR-33b-3p |  |  | x |  |
| hsa-miR-33b-5p * | x | x | x | x |
| hsa-miR-340-3p | x | x | x |  |
| hsa-miR-340-5p |  | x | x |  |
| hsa-miR-342-5p |  |  | x | x |
| hsa-miR-345-5p |  |  | x |  |
| hsa-miR-346 |  |  | x | x |
| hsa-miR-34a-3p | x |  | x |  |
| hsa-miR-34a-5p |  |  | x |  |
| hsa-miR-34b-3p | x |  | x |  |
| hsa-miR-34b-5p |  | x | x |  |
| hsa-miR-34c-3p |  |  | x |  |
| hsa-miR-34c-5p |  | x | x |  |
| hsa-miR-3605-3p |  |  | x |  |
| hsa-miR-3605-5p |  |  | x |  |
| hsa-miR-3606-5p |  |  | x |  |
| hsa-miR-3607-3p |  |  | x |  |
| hsa-miR-3609 |  |  | x |  |
| hsa-miR-3610 |  |  | x |  |
| hsa-miR-3611 |  |  | x |  |
| hsa-miR-3612 |  |  | x |  |
| hsa-miR-3613-3p |  |  | x |  |
| hsa-miR-3613-5p |  |  | x |  |
| hsa-miR-361-3p | x | x | x | x |
| hsa-miR-3614-3p |  |  | x |  |
| hsa-miR-3614-5p |  |  | x |  |
| hsa-miR-3615 |  |  | x |  |
| hsa-miR-361-5p |  |  | x |  |
| hsa-miR-3616-3p |  |  | x |  |
| hsa-miR-3616-5p |  |  | x |  |
| hsa-miR-3617-5p |  |  | x |  |
| hsa-miR-3619 |  |  | x |  |
| hsa-miR-3619-5p |  |  | x |  |
| hsa-miR-3621 |  |  | x |  |
| hsa-miR-3622a-3p |  |  | x |  |
| hsa-miR-3622b-3p |  |  | x |  |
| hsa-miR-3622b-5p |  |  | x |  |
| hsa-miR-362-3p |  |  | x |  |
| hsa-miR-362-5p |  | x | x |  |
| hsa-miR-363-3p |  |  | x | x |
| hsa-miR-363-5p |  |  | x | x |
| hsa-miR-3646 |  |  | x |  |
| hsa-miR-3649 |  |  | x |  |
| hsa-miR-3650 |  |  | x |  |
| hsa-miR-3651 |  |  | x |  |
| hsa-miR-3652 |  |  | x |  |
| hsa-miR-3654 |  |  | x |  |
| hsa-miR-3655 |  |  | x |  |
| hsa-miR-3656 |  |  | x |  |
| hsa-miR-3657 |  |  | x |  |
| hsa-miR-3658 |  |  | x |  |
| hsa-miR-3659 |  |  | x |  |
| hsa-miR-365a-3p | x |  | x |  |
| hsa-miR-365b-5p |  |  | x |  |
| hsa-miR-3660 |  |  | x |  |
| hsa-miR-3661 |  |  | x |  |
| hsa-miR-3662 |  |  | x |  |
| hsa-miR-3663-5p |  |  | x |  |
| hsa-miR-3664-5p |  |  | x |  |
| hsa-miR-3666 |  |  | x |  |
| hsa-miR-3667-3p |  |  | x |  |
| hsa-miR-3667-5p |  |  | x |  |
| hsa-miR-3668 |  |  | x |  |
| hsa-miR-3669 |  |  | x |  |
| hsa-miR-3670 |  |  | x |  |
| hsa-miR-3671 |  |  | x |  |
| hsa-miR-3672 |  |  | x |  |
| hsa-miR-3673 |  |  | x |  |
| hsa-miR-367-3p |  | x | x | x |
| hsa-miR-3674 |  |  | x |  |
| hsa-miR-3675-3p |  |  | x |  |
| hsa-miR-3675-5p |  |  | x |  |
| hsa-miR-367-5p | x | x | x | x |
| hsa-miR-3678-5p |  |  | x |  |
| hsa-miR-3679-3p |  |  | x |  |
| hsa-miR-3679-5p |  |  | x |  |
| hsa-miR-368 | x |  |  |  |
| hsa-miR-3680-3p |  |  | x |  |
| hsa-miR-3680-5p |  |  | x |  |
| hsa-miR-3681-3p |  |  | x |  |
| hsa-miR-3681-5p |  |  | x |  |
| hsa-miR-3682-3p |  |  | x |  |
| hsa-miR-3683 |  |  | x |  |
| hsa-miR-3684 |  |  | x |  |
| hsa-miR-3685 |  |  | x |  |
| hsa-miR-3686 |  |  | x |  |
| hsa-miR-3687 |  |  | x |  |
| hsa-miR-3688-3p |  |  | x |  |
| hsa-miR-3689a-3p |  |  | x |  |
| hsa-miR-3689a-5p |  |  | x |  |
| hsa-miR-3689f |  |  | x |  |
| hsa-miR-3691-3p |  |  | x |  |
| hsa-miR-3691-5p |  |  | x |  |
| hsa-miR-3692-3p |  |  | x |  |
| hsa-miR-3692-5p |  |  | x |  |
| hsa-miR-369-3p * |  | x | x | x |
| hsa-miR-369-5p |  | x | x | x |
| hsa-miR-370-3p * |  |  | x | x |
| hsa-miR-3713 |  |  | x |  |
| hsa-miR-3714 |  |  | x |  |
| hsa-miR-371a-3p * |  |  | x | x |
| hsa-miR-371a-5p |  |  | x |  |
| hsa-miR-371b-5p |  |  | x |  |
| hsa-miR-372-3p |  | x | x |  |
| hsa-miR-373-3p |  | x | x |  |
| hsa-miR-373-5p |  |  | x |  |
| hsa-miR-374a-3p | x | x | x | x |
| hsa-miR-374a-5p |  | x | x |  |
| hsa-miR-374b-3p | x | x | x | x |
| hsa-miR-374b-5p |  |  | x |  |
| hsa-miR-374c-5p |  |  | x |  |
| hsa-miR-375 |  | x | x |  |
| hsa-miR-376a-3p |  |  | x |  |
| hsa-miR-376a-5p | x | x | x | x |
| hsa-miR-376b-3p |  |  | x |  |
| hsa-miR-376c-3p |  |  | x |  |
| hsa-miR-377-3p * |  |  | x | x |
| hsa-miR-377-5p | x | x | x | x |
| hsa-miR-378a-3p |  |  | x |  |
| hsa-miR-378a-5p |  |  | x |  |
| hsa-miR-378b |  |  | x |  |
| hsa-miR-378c |  |  | x |  |
| hsa-miR-378d |  |  | x |  |
| hsa-miR-378g |  |  | x |  |
| hsa-miR-379-3p | x |  | x |  |
| hsa-miR-379-5p |  |  |  | x |
| hsa-miR-380-3p * |  | x | x | x |
| hsa-miR-380-5p |  | x | x | x |
| hsa-miR-381-3p |  | x | x | x |
| hsa-miR-382-5p |  | x | x | x |
| hsa-miR-383-5p |  | x | x | x |
| hsa-miR-384 |  | x | x | x |
| hsa-miR-3907 |  |  | x |  |
| hsa-miR-3908 |  |  | x |  |
| hsa-miR-3909 |  |  | x |  |
| hsa-miR-3910 |  |  | x |  |
| hsa-miR-3911 |  |  | x |  |
| hsa-miR-3912-3p |  |  | x |  |
| hsa-miR-3913-5p |  |  | x |  |
| hsa-miR-3914 |  |  | x |  |
| hsa-miR-3915 |  |  | x |  |
| hsa-miR-3916 |  |  | x |  |
| hsa-miR-3918 |  |  | x |  |
| hsa-miR-3919 |  |  | x |  |
| hsa-miR-3920 |  |  | x |  |
| hsa-miR-3921 |  |  | x |  |
| hsa-miR-3922-3p |  |  | x |  |
| hsa-miR-3923 |  |  | x |  |
| hsa-miR-3925-3p |  |  | x |  |
| hsa-miR-3925-5p |  |  | x |  |
| hsa-miR-3927-3p |  |  | x |  |
| hsa-miR-3928-3p |  |  | x |  |
| hsa-miR-3929 |  |  | x |  |
| hsa-miR-3934-5p |  |  | x |  |
| hsa-miR-3935 |  |  | x |  |
| hsa-miR-3936 |  |  | x |  |
| hsa-miR-3937 |  |  | x |  |
| hsa-miR-3938 |  |  | x |  |
| hsa-miR-3940-5p |  |  | x |  |
| hsa-miR-3941 |  |  | x |  |
| hsa-miR-3942-5p |  |  | x |  |
| hsa-miR-3944-3p |  |  | x |  |
| hsa-miR-3945 |  |  | x |  |
| hsa-miR-409-3p | x | x | x |  |
| hsa-miR-409-5p |  |  | x | x |
| hsa-miR-410-3p |  |  | x |  |
| hsa-miR-411-3p |  |  | x | x |
| hsa-miR-411-5p |  | x | x |  |
| hsa-miR-412-3p |  |  | x | x |
| hsa-miR-421 |  |  | x |  |
| hsa-miR-422a |  |  | x |  |
| hsa-miR-423-3p |  |  | x |  |
| hsa-miR-424-3p | x | x | x | x |
| hsa-miR-424-5p |  |  | x |  |
| hsa-miR-4251 |  |  | x |  |
| hsa-miR-4252 |  |  | x |  |
| hsa-miR-4253 |  |  | x |  |
| hsa-miR-425-3p |  |  | x | x |
| hsa-miR-4254 |  |  | x |  |
| hsa-miR-425-5p |  |  |  | x |
| hsa-miR-4256 |  |  | x |  |
| hsa-miR-4257 |  |  | x |  |
| hsa-miR-4258 |  |  | x |  |
| hsa-miR-4259 |  |  | x |  |
| hsa-miR-4260 |  |  | x |  |
| hsa-miR-4261 |  |  | x |  |
| hsa-miR-4262 |  |  | x |  |
| hsa-miR-4263 |  |  | x |  |
| hsa-miR-4264 |  |  | x |  |
| hsa-miR-4265 |  |  | x |  |
| hsa-miR-4266 |  |  | x |  |
| hsa-miR-4267 |  |  | x |  |
| hsa-miR-4268 |  |  | x |  |
| hsa-miR-4272 |  |  | x |  |
| hsa-miR-4273 |  |  | x |  |
| hsa-miR-4274 |  |  | x |  |
| hsa-miR-4275 |  |  | x |  |
| hsa-miR-4276 |  |  | x |  |
| hsa-miR-4277 |  |  | x |  |
| hsa-miR-4278 |  |  | x |  |
| hsa-miR-4279 |  |  | x |  |
| hsa-miR-4280 |  |  | x |  |
| hsa-miR-4282 |  |  | x |  |
| hsa-miR-4283 |  |  | x |  |
| hsa-miR-4284 |  |  | x |  |
| hsa-miR-4285 |  |  | x |  |
| hsa-miR-4287 |  |  | x |  |
| hsa-miR-4288 |  |  | x |  |
| hsa-miR-4289 |  |  | x |  |
| hsa-miR-429 ^b^ | x | x | x | x |
| hsa-miR-4290 |  |  | x |  |
| hsa-miR-4292 |  |  | x |  |
| hsa-miR-4294 |  |  | x |  |
| hsa-miR-4295 |  |  | x |  |
| hsa-miR-4297 |  |  | x |  |
| hsa-miR-4299 |  |  | x |  |
| hsa-miR-4300 |  |  | x |  |
| hsa-miR-4301 |  |  | x |  |
| hsa-miR-4302 |  |  | x |  |
| hsa-miR-4304 |  |  | x |  |
| hsa-miR-4305 |  |  | x |  |
| hsa-miR-4306 |  |  | x |  |
| hsa-miR-4307 |  |  | x |  |
| hsa-miR-4308 |  |  | x |  |
| hsa-miR-4309 |  |  | x |  |
| hsa-miR-4310 |  |  | x |  |
| hsa-miR-4311 |  |  | x |  |
| hsa-miR-4312 |  |  | x |  |
| hsa-miR-431-3p | x | x | x | x |
| hsa-miR-4314 |  |  | x |  |
| hsa-miR-4315 |  |  | x |  |
| hsa-miR-431-5p | x | x |  | x |
| hsa-miR-4316 |  |  | x |  |
| hsa-miR-4317 |  |  | x |  |
| hsa-miR-4318 |  |  | x |  |
| hsa-miR-4319 |  |  | x |  |
| hsa-miR-4320 |  |  | x |  |
| hsa-miR-4321 |  |  | x |  |
| hsa-miR-4322 |  |  | x |  |
| hsa-miR-4323 |  |  | x |  |
| hsa-miR-432-3p | x | x | x | x |
| hsa-miR-4324 |  |  | x |  |
| hsa-miR-4325 |  |  | x |  |
| hsa-miR-432-5p |  | x | x |  |
| hsa-miR-4326 |  |  | x |  |
| hsa-miR-4327 |  |  | x |  |
| hsa-miR-4328 |  |  | x |  |
| hsa-miR-4329 |  |  | x |  |
| hsa-miR-4330 |  |  | x |  |
| hsa-miR-433-3p |  | x | x |  |
| hsa-miR-4428 |  |  | x |  |
| hsa-miR-4436a |  |  | x |  |
| hsa-miR-4436b-3p |  |  | x |  |
| hsa-miR-4438 |  |  | x |  |
| hsa-miR-4445-3p |  |  | x |  |
| hsa-miR-4446-5p |  |  | x |  |
| hsa-miR-4447 |  |  | x |  |
| hsa-miR-4452 |  |  | x |  |
| hsa-miR-4453 |  |  | x |  |
| hsa-miR-4455 |  |  | x |  |
| hsa-miR-4462 |  |  | x |  |
| hsa-miR-4469 |  |  | x |  |
| hsa-miR-4472 |  |  | x |  |
| hsa-miR-448 | x | x | x | x |
| hsa-miR-4493 |  |  | x |  |
| hsa-miR-4495 |  |  | x |  |
| hsa-miR-4498 |  |  | x |  |
| hsa-miR-449a |  | x | x |  |
| hsa-miR-449b-3p |  |  | x |  |
| hsa-miR-449b-5p |  | x | x |  |
| hsa-miR-449c-3p | x |  | x |  |
| hsa-miR-449c-5p | x | x | x |  |
| hsa-miR-4502 |  |  | x |  |
| hsa-miR-4503 |  |  | x |  |
| hsa-miR-4505 |  |  | x |  |
| hsa-miR-450a-5p |  | x | x |  |
| hsa-miR-450b-3p |  | x | x | x |
| hsa-miR-450b-5p |  | x | x | x |
| hsa-miR-452-3p |  | x | x | x |
| hsa-miR-4524a-5p |  |  | x |  |
| hsa-miR-452-5p |  | x | x |  |
| hsa-miR-4529-3p |  |  | x |  |
| hsa-miR-4540 |  |  | x |  |
| hsa-miR-454-3p |  | x | x |  |
| hsa-miR-454-5p | x | x | x | x |
| hsa-miR-455-3p |  |  | x | x |
| hsa-miR-455-5p |  | x | x |  |
| hsa-miR-4640-5p |  |  | x |  |
| hsa-miR-4649-5p |  |  | x |  |
| hsa-miR-4653-3p |  |  | x |  |
| hsa-miR-4659b-5p |  |  | x |  |
| hsa-miR-4662a-3p |  |  | x |  |
| hsa-miR-4667-3p |  |  | x |  |
| hsa-miR-4667-5p |  |  | x |  |
| hsa-miR-4678 |  |  | x |  |
| hsa-miR-4682 |  |  | x |  |
| hsa-miR-4684-3p |  |  | x |  |
| hsa-miR-4690-5p |  |  | x |  |
| hsa-miR-4692 |  |  | x |  |
| hsa-miR-4693-3p |  |  | x |  |
| hsa-miR-4695-3p |  |  | x |  |
| hsa-miR-4700-3p |  |  | x |  |
| hsa-miR-4704-5p |  |  | x |  |
| hsa-miR-4708-3p |  |  | x |  |
| hsa-miR-4708-5p |  |  | x |  |
| hsa-miR-4717-5p |  |  | x |  |
| hsa-miR-4727-3p |  |  | x |  |
| hsa-miR-4727-5p |  |  | x |  |
| hsa-miR-4733-5p |  |  | x |  |
| hsa-miR-4747-5p |  |  | x |  |
| hsa-miR-4756-3p |  |  | x |  |
| hsa-miR-4758-3p |  |  | x |  |
| hsa-miR-4759 |  |  | x |  |
| hsa-miR-4760-3p |  |  | x |  |
| hsa-miR-4764-5p |  |  | x |  |
| hsa-miR-4765 |  |  | x |  |
| hsa-miR-4769-3p |  |  | x |  |
| hsa-miR-4777-3p |  |  | x |  |
| hsa-miR-4783-3p |  |  | x |  |
| hsa-miR-4784 |  |  | x |  |
| hsa-miR-4787-5p |  |  | x |  |
| hsa-miR-4799-3p |  |  | x |  |
| hsa-miR-4800-3p |  |  | x |  |
| hsa-miR-4804-3p |  |  | x |  |
| hsa-miR-483-3p |  |  | x |  |
| hsa-miR-484 |  |  | x |  |
| hsa-miR-485-3p |  |  | x | x |
| hsa-miR-485-5p |  |  | x | x |
| hsa-miR-486-5p |  |  |  | x |
| hsa-miR-487a-3p |  |  | x | x |
| hsa-miR-487b-3p |  |  | x | x |
| hsa-miR-488-3p |  | x | x | x |
| hsa-miR-488-5p |  |  | x |  |
| hsa-miR-490-3p |  | x | x | x |
| hsa-miR-490-5p | x | x |  |  |
| hsa-miR-491-3p |  |  | x | x |
| hsa-miR-492 | x |  | x | x |
| hsa-miR-493-3p |  | x | x | x |
| hsa-miR-493-5p | x | x | x |  |
| hsa-miR-494-3p |  | x | x |  |
| hsa-miR-496 |  |  |  | x |
| hsa-miR-497-3p |  |  | x | x |
| hsa-miR-498 |  |  | x |  |
| hsa-miR-499a-3p |  |  | x | x |
| hsa-miR-499a-5p |  |  |  | x |
| hsa-miR-500a-3p |  |  | x |  |
| hsa-miR-500b-5p |  |  | x |  |
| hsa-miR-501-3p |  | x | x |  |
| hsa-miR-501-5p |  |  | x | x |
| hsa-miR-502-3p |  |  | x |  |
| hsa-miR-502-5p |  | x | x | x |
| hsa-miR-503-5p |  |  | x | x |
| hsa-miR-504-5p |  |  | x | x |
| hsa-miR-505-3p |  | x | x |  |
| hsa-miR-505-5p |  |  | x |  |
| hsa-miR-506-3p |  |  | x | x |
| hsa-miR-507 | x | x | x | x |
| hsa-miR-508-3p |  | x | x | x |
| hsa-miR-508-5p | x |  | x | x |
| hsa-miR-509-3-5p | x | x | x | x |
| hsa-miR-509-3p |  | x | x |  |
| hsa-miR-510-5p |  |  | x | x |
| hsa-miR-511-5p |  | x | x |  |
| hsa-miR-512-3p |  | x | x | x |
| hsa-miR-512-5p |  |  | x | x |
| hsa-miR-513-5p |  |  | x | x |
| hsa-miR-513a-3p |  | x | x |  |
| hsa-miR-513b-5p | x |  | x | x |
| hsa-miR-513c-5p | x | x | x | x |
| hsa-miR-514b-3p |  |  | x |  |
| hsa-miR-514b-5p |  |  | x |  |
| hsa-miR-515-3p |  | x | x |  |
| hsa-miR-515-5p |  |  |  | x |
| hsa-miR-516a-3p | x | x | x |  |
| hsa-miR-516a-5p |  |  |  | x |
| hsa-miR-516b-5p |  |  | x |  |
| hsa-miR-517-5p | x |  | x | x |
| hsa-miR-517a-3p |  | x | x | x |
| hsa-miR-517b-3p |  | x | x |  |
| hsa-miR-517c-3p |  | x | x | x |
| hsa-miR-518a-3p |  | x | x | x |
| hsa-miR-518a-5p |  | x |  | x |
| hsa-miR-518b |  |  | x | x |
| hsa-miR-518c-3p |  | x | x | x |
| hsa-miR-518c-5p |  |  | x | x |
| hsa-miR-518d-3p |  | x | x |  |
| hsa-miR-518e-3p |  |  | x |  |
| hsa-miR-518e-5p | x | x | x | x |
| hsa-miR-518f-3p |  | x | x | x |
| hsa-miR-518f-5p | x | x |  | x |
| hsa-miR-519a-3p |  | x | x | x |
| hsa-miR-519b-3p | x | x | x | x |
| hsa-miR-519c-3p |  |  | x | x |
| hsa-miR-519d-3p |  |  | x |  |
| hsa-miR-519e-3p |  |  | x | x |
| hsa-miR-519e-5p |  |  | x | x |
| hsa-miR-520a-3p |  | x | x | x |
| hsa-miR-520a-5p |  | x | x | x |
| hsa-miR-520b |  | x | x |  |
| hsa-miR-520c-3p |  | x | x |  |
| hsa-miR-520c-5p | x | x | x | x |
| hsa-miR-520d-3p |  | x | x |  |
| hsa-miR-520d-5p |  |  | x |  |
| hsa-miR-520e |  | x | x | x |
| hsa-miR-520f-3p |  | x | x |  |
| hsa-miR-520g-3p |  | x | x | x |
| hsa-miR-520h |  | x | x |  |
| hsa-miR-521 |  |  | x | x |
| hsa-miR-522-3p |  | x | x |  |
| hsa-miR-523-3p |  | x | x |  |
| hsa-miR-524-3p | x | x | x | x |
| hsa-miR-524-5p | x |  | x | x |
| hsa-miR-525-3p |  | x | x | x |
| hsa-miR-525-5p |  |  |  | x |
| hsa-miR-526b-3p | x | x | x |  |
| hsa-miR-526b-5p |  | x | x | x |
| hsa-miR-532-3p |  |  | x |  |
| hsa-miR-539-5p |  | x | x |  |
| hsa-miR-541-3p |  |  | x | x |
| hsa-miR-541-5p | x |  | x | x |
| hsa-miR-542-3p |  |  | x |  |
| hsa-miR-542-5p |  |  | x | x |
| hsa-miR-543 | x | x | x | x |
| hsa-miR-544a |  | x | x | x |
| hsa-miR-544b |  |  | x |  |
| hsa-miR-545-3p |  | x | x |  |
| hsa-miR-545-5p | x | x | x | x |
| hsa-miR-548a-3p |  |  | x |  |
| hsa-miR-548a-5p ^b^ | x | x | x | x |
| hsa-miR-548aa |  |  | x |  |
| hsa-miR-548aj-3p |  |  | x |  |
| hsa-miR-548al |  |  | x |  |
| hsa-miR-548an |  |  | x |  |
| hsa-miR-548b-3p | x |  | x | x |
| hsa-miR-548b-5p |  | x | x |  |
| hsa-miR-548c-3p |  |  | x |  |
| hsa-miR-548c-5p |  | x | x |  |
| hsa-miR-548d-3p |  |  | x | x |
| hsa-miR-548d-5p |  | x | x |  |
| hsa-miR-548e-3p | x | x | x | x |
| hsa-miR-548f-5p | x | x | x |  |
| hsa-miR-548g-3p | x | x | x | x |
| hsa-miR-548h-5p | x | x | x |  |
| hsa-miR-548i | x | x | x | x |
| hsa-miR-548j-5p | x | x | x | x |
| hsa-miR-548k | x | x | x | x |
| hsa-miR-548l | x | x | x | x |
| hsa-miR-548m | x | x | x | x |
| hsa-miR-548n | x | x | x | x |
| hsa-miR-548o-3p | x | x | x |  |
| hsa-miR-548p | x | x | x | x |
| hsa-miR-548q |  |  | x |  |
| hsa-miR-548t-5p |  |  | x |  |
| hsa-miR-548u |  |  | x |  |
| hsa-miR-548v |  |  | x |  |
| hsa-miR-548w |  |  | x |  |
| hsa-miR-548x-3p |  |  | x |  |
| hsa-miR-548y |  |  | x |  |
| hsa-miR-548z |  |  | x |  |
| hsa-miR-549a | x | x | x | x |
| hsa-miR-550a-3p |  |  |  | x |
| hsa-miR-550a-5p |  |  |  | x |
| hsa-miR-550b-3p |  |  | x |  |
| hsa-miR-551a |  |  | x | x |
| hsa-miR-551b-3p |  |  | x |  |
| hsa-miR-551b-5p |  |  | x |  |
| hsa-miR-552-3p |  | x | x | x |
| hsa-miR-553 | x | x | x | x |
| hsa-miR-554 | x |  | x | x |
| hsa-miR-555 | x | x | x | x |
| hsa-miR-556-3p |  | x | x | x |
| hsa-miR-556-5p | x | x | x | x |
| hsa-miR-557 | x |  | x | x |
| hsa-miR-558 |  |  | x | x |
| hsa-miR-559 |  |  | x | x |
| hsa-miR-561-3p | x | x | x | x |
| hsa-miR-562 | x | x | x | x |
| hsa-miR-563 | x | x | x | x |
| hsa-miR-564 |  |  | x | x |
| hsa-miR-566 |  |  | x | x |
| hsa-miR-567 | x | x | x | x |
| hsa-miR-569 | x |  | x | x |
| hsa-miR-570-3p |  |  | x |  |
| hsa-miR-571 | x | x | x | x |
| hsa-miR-572 |  | x | x |  |
| hsa-miR-573 | x | x | x | x |
| hsa-miR-575 |  |  | x | x |
| hsa-miR-576-3p |  |  | x |  |
| hsa-miR-576-5p |  |  | x | x |
| hsa-miR-577 | x | x | x | x |
| hsa-miR-578 | x | x | x | x |
| hsa-miR-579 |  | x | x | x |
| hsa-miR-580 | x | x | x | x |
| hsa-miR-581 |  | x | x | x |
| hsa-miR-582-3p |  |  | x | x |
| hsa-miR-582-5p | x | x | x | x |
| hsa-miR-583 |  |  | x | x |
| hsa-miR-584-5p | x |  | x | x |
| hsa-miR-585 | x | x | x | x |
| hsa-miR-586 | x | x | x | x |
| hsa-miR-587 | x | x | x | x |
| hsa-miR-588 |  | x | x | x |
| hsa-miR-589-3p | x |  | x | x |
| hsa-miR-589-5p |  |  | x | x |
| hsa-miR-590-3p | x | x | x |  |
| hsa-miR-590-5p |  | x | x |  |
| hsa-miR-591 | x | x | x | x |
| hsa-miR-592 | x | x | x |  |
| hsa-miR-593-3p | x |  | x | x |
| hsa-miR-593-5p |  |  |  | x |
| hsa-miR-595 | x |  | x | x |
| hsa-miR-596 | x |  | x | x |
| hsa-miR-597 |  | x | x |  |
| hsa-miR-598 |  | x | x |  |
| hsa-miR-599 | x | x | x | x |
| hsa-miR-600 | x |  | x | x |
| hsa-miR-601 |  |  | x |  |
| hsa-miR-602 |  |  | x |  |
| hsa-miR-603 | x |  | x |  |
| hsa-miR-604 |  |  | x |  |
| hsa-miR-605 | x |  | x | x |
| hsa-miR-606 |  |  | x | x |
| hsa-miR-607 |  |  | x | x |
| hsa-miR-608 | x |  | x | x |
| hsa-miR-609 | x |  | x | x |
| hsa-miR-610 | x |  | x |  |
| hsa-miR-612 |  |  | x |  |
| hsa-miR-613 | x | x | x | x |
| hsa-miR-614 | x | x | x | x |
| hsa-miR-615-3p |  |  | x | x |
| hsa-miR-615-5p | x |  | x | x |
| hsa-miR-616-3p |  | x | x | x |
| hsa-miR-616-5p |  |  | x | x |
| hsa-miR-617 | x | x | x | x |
| hsa-miR-618 |  | x | x | x |
| hsa-miR-619-3p | x | x | x |  |
| hsa-miR-620 |  |  | x | x |
| hsa-miR-621 | x | x | x | x |
| hsa-miR-622 | x | x | x |  |
| hsa-miR-623 | x | x | x |  |
| hsa-miR-624-3p | x |  | x | x |
| hsa-miR-624-5p | x | x | x | x |
| hsa-miR-625-5p |  |  | x |  |
| hsa-miR-626 | x | x | x | x |
| hsa-miR-627 |  |  | x |  |
| hsa-miR-628-3p |  |  | x |  |
| hsa-miR-628-5p |  | x | x |  |
| hsa-miR-629-3p |  | x | x |  |
| hsa-miR-629-5p |  |  | x |  |
| hsa-miR-630 |  | x | x | x |
| hsa-miR-631 | x |  | x | x |
| hsa-miR-632 | x |  | x |  |
| hsa-miR-633 |  |  | x | x |
| hsa-miR-634 | x |  | x | x |
| hsa-miR-635 |  |  | x | x |
| hsa-miR-636 |  |  | x |  |
| hsa-miR-637 |  |  | x | x |
| hsa-miR-639 |  | x | x |  |
| hsa-miR-640 | x |  | x | x |
| hsa-miR-641 |  |  | x | x |
| hsa-miR-642a-5p |  |  | x |  |
| hsa-miR-642b-3p |  |  | x |  |
| hsa-miR-643 | x | x | x | x |
| hsa-miR-644 | x | x | x | x |
| hsa-miR-645 | x |  | x |  |
| hsa-miR-646 | x | x |  |  |
| hsa-miR-647 |  |  | x | x |
| hsa-miR-648 | x | x | x | x |
| hsa-miR-649 | x | x | x | x |
| hsa-miR-651 |  | x | x |  |
| hsa-miR-653 |  |  | x | x |
| hsa-miR-654-3p |  |  | x | x |
| hsa-miR-654-5p |  |  |  | x |
| hsa-miR-655 |  | x | x | x |
| hsa-miR-656 |  |  | x | x |
| hsa-miR-657 |  |  | x | x |
| hsa-miR-658 | x |  | x | x |
| hsa-miR-659-3p |  |  | x |  |
| hsa-miR-660-5p |  |  | x |  |
| hsa-miR-661 | x |  | x |  |
| hsa-miR-662 | x |  | x | x |
| hsa-miR-663b | x |  | x |  |
| hsa-miR-664a-3p |  |  | x | x |
| hsa-miR-664a-5p |  |  | x |  |
| hsa-miR-665 | x |  | x | x |
| hsa-miR-668 |  |  | x | x |
| hsa-miR-670-5p | x | x | x |  |
| hsa-miR-671-3p |  |  | x | x |
| hsa-miR-671-5p |  |  | x |  |
| hsa-miR-672 |  |  |  | x |
| hsa-miR-674 |  |  | x | x |
| hsa-miR-675-3p |  |  | x |  |
| hsa-miR-675-5p |  |  | x |  |
| hsa-miR-676-3p |  |  | x |  |
| hsa-miR-676-5p |  |  | x |  |
| hsa-miR-708-3p | x | x | x |  |
| hsa-miR-708-5p |  | x | x |  |
| hsa-miR-7-1-3p |  |  |  | x |
| hsa-miR-7-2-3p |  |  | x | x |
| hsa-miR-744-3p |  |  | x | x |
| hsa-miR-758-3p |  |  | x | x |
| hsa-miR-759 | x | x | x |  |
| hsa-miR-760 |  |  | x |  |
| hsa-miR-764 |  |  | x |  |
| hsa-miR-765 |  |  | x | x |
| hsa-miR-767-3p | x |  | x |  |
| hsa-miR-767-5p | x | x | x |  |
| hsa-miR-769-3p | x | x | x | x |
| hsa-miR-769-5p | x | x | x | x |
| hsa-miR-770-5p |  |  | x | x |
| hsa-miR-802 | x | x | x | x |
| hsa-miR-871 |  |  |  | x |
| hsa-miR-872 | x |  | x | x |
| hsa-miR-873-5p |  | x | x | x |
| hsa-miR-875-3p | x |  | x | x |
| hsa-miR-875-5p | x | x | x | x |
| hsa-miR-876-3p |  | x | x | x |
| hsa-miR-876-5p |  | x | x | x |
| hsa-miR-877-3p |  |  | x |  |
| hsa-miR-885-3p |  |  | x | x |
| hsa-miR-885-5p |  |  | x |  |
| hsa-miR-886-3p |  | x |  |  |
| hsa-miR-887 |  |  | x | x |
| hsa-miR-888-3p | x |  | x | x |
| hsa-miR-888-5p | x |  | x |  |
| hsa-miR-889 |  |  | x | x |
| hsa-miR-890 |  | x | x | x |
| hsa-miR-891a |  |  | x | x |
| hsa-miR-891b | x | x | x | x |
| hsa-miR-892a |  | x | x | x |
| hsa-miR-892b | x |  | x | x |
| hsa-miR-920 |  |  | x | x |
| hsa-miR-921 |  |  | x | x |
| hsa-miR-922 |  |  |  | x |
| hsa-miR-924 | x | x | x | x |
| hsa-miR-92a-1-5p | x | x | x |  |
| hsa-miR-92a-2-5p |  |  | x | x |
| hsa-miR-92b-5p |  |  | x | x |
| hsa-miR-933 |  |  | x | x |
| hsa-miR-93-3p | x |  | x |  |
| hsa-miR-934 | x | x | x | x |
| hsa-miR-935 | x |  | x | x |
| hsa-miR-936 |  |  | x | x |
| hsa-miR-937-3p |  |  | x | x |
| hsa-miR-938 | x | x | x | x |
| hsa-miR-939-5p |  |  | x |  |
| hsa-miR-9-3p |  | x | x |  |
| hsa-miR-941 | x | x | x | x |
| hsa-miR-942-5p | x | x | x |  |
| hsa-miR-943 | x |  | x | x |
| hsa-miR-944 | x | x | x |  |
| hsa-miR-95-3p |  | x | x |  |
| hsa-miR-9-5p |  | x | x | x |
| hsa-miR-96-3p | x | x | x | x |
| hsa-miR-96-5p |  |  |  | x |
| hsa-miR-98-5p * |  | x | x | x |
| hsa-miR-99a-3p | x | x | x | x |
| hsa-miR-99a-5p |  | x | x |  |
| hsa-miR-99b-3p | x | x | x |  |
| hsa-miR-99b-5p |  |  | x |  |

*Note:* miRNAs absent in urine or ocular fluid are indicated with ^*^.
